# Supplementary figures and images for: Seroprevalence of SARS-CoV-2 infection in Cincinnati Ohio USA from August to December 2020
Source: PLoS One. 2021 Jul 14;16(7):e0254667. doi: 10.1371/journal.pone.0254667 (PMC8279307; doi:10.1371/journal.pone.0254667)

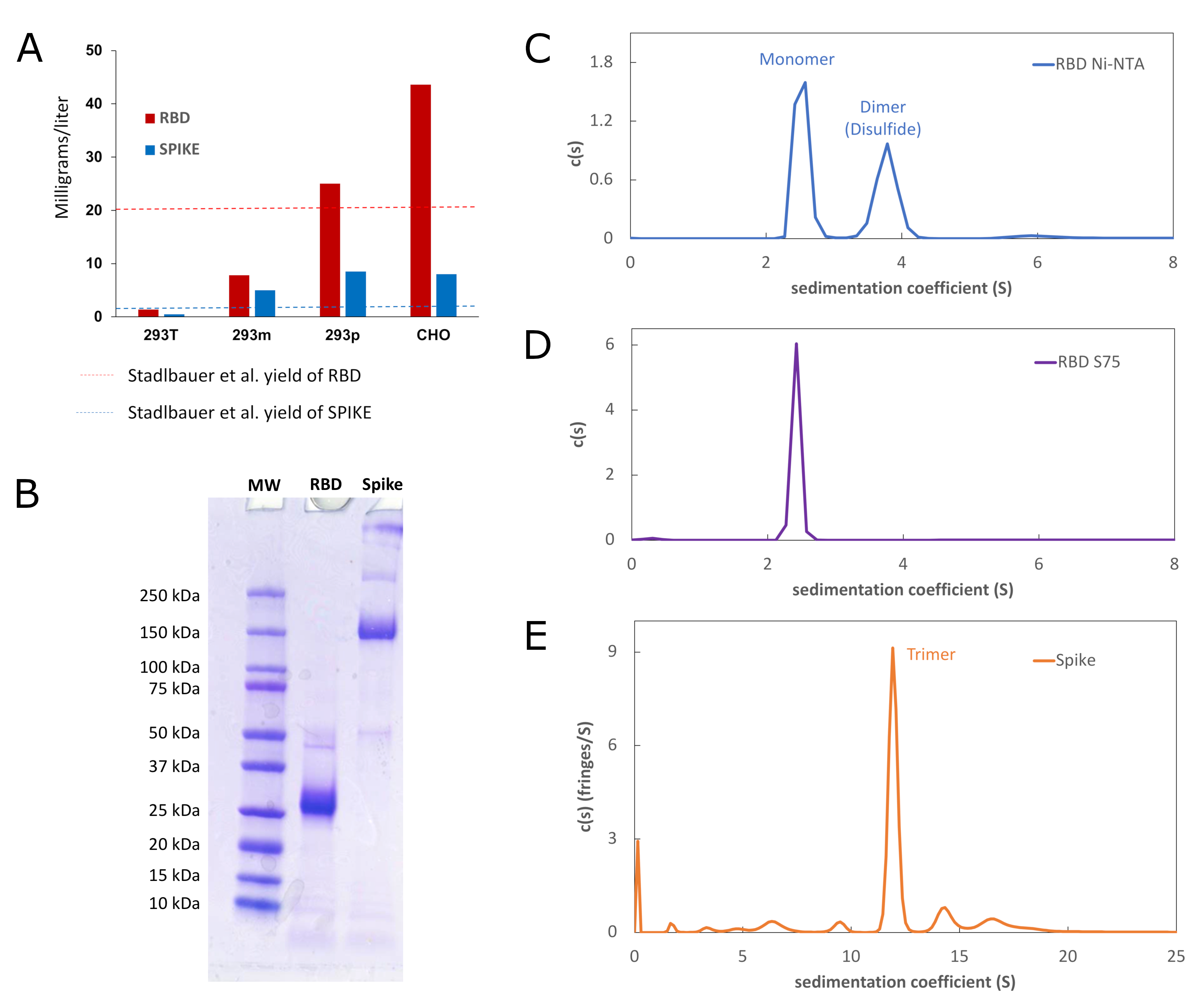

Supplement: S1 Fig — A) Comparison of expression yields in different cell lines. Dashed lines represent the reported yields from Stadlbauer et al. [10]. B) Reducing SDS-PAGE gel showing RBD purified by Ni-NTA and gel filtration chromatography and Spike protein purified by Ni-NTA chromatography. The full gel image is shown in this panel so there is no additional need to provide a separate Supporting Information file with this exact same full gel image. C-E) Sedimentation velocity analytical ultracentrifugation analysis of protein quality and assembly state in solution. C) Sedimentation coefficient distribution for RBD purified by Ni-NTA revealing monomer and disulfide-linked dimer species. D) Sedimentation coefficient distribution for monomeric RBD (experimental MW of 31.1 kDa) purified by S75 gel filtration. E) Sedimentation coefficient distribution for Spike protein showing that trimer is the predominant species (experimental MW of ~519 kDa). The trimer sedimentation coefficient of 12.9 S was consistent with the value (12.6 S) calculated by HullRad [28] hydrodynamic modeling of the glycosylated spike trimer structure (PDB 6VXX). The S protein monomer is predicted to sediment at approximately 5.5 S, but the exact value will depend on the hydrodynamic shape of the isolated monomeric species. (TIF) [file pone.0254667.s001.tif]
