# Supplementary material for: Seroprevalence of SARS-CoV-2 infection in Cincinnati Ohio USA from August to December 2020
Source: PLoS One. 2021 Jul 14;16(7):e0254667. doi: 10.1371/journal.pone.0254667 (PMC8279307; doi:10.1371/journal.pone.0254667)
Supplement: Attachment — Submitted filename: Editorial Revisions2.pdf [file pone.0254667.s003.pdf]

1. Please ensure that your manuscript meets PLOS ONE's style requirements, including those for file naming. The PLOS ONE style templates can be found at

https://journals.plos.org/plosone/s/file?id=wjVg/PLOSOne_formatting_sample_main_body.pdf and

https://journals.plos.org/plosone/s/file?id=ba62/PLOSOne_formatting_sample_title_authors_affiliations.pdf

Done

2. In your Methods section, please provide additional information about the biological samples and the demographic details of the donors. Please ensure you have provided sufficient details to replicate the analyses such as:

a) the date(s) researchers accessed the samples,

To address this, we added information to lines 118-120

b) the date range that samples were originally collected,

and the date range the samples were collected was added to line 118-119.

c) a description of any inclusion/exclusion criteria that were applied to sample selection,

We excluded some samples for this study and added "Samples without complete biogeographical data were excluded" to lines 129-131 to address this, and samples that were duplicate donors.

d) a table of relevant demographic details of donors (if disclosed), \\.

We have included a Supporting Information table as an Excel spreadsheet of relevant demographic information sufficient to reproduce the data in this publication. While we have excluded zip code from the demographic data, we have replaced it with a more general region location to further de-identify the information that still allows full reproducibility of our analyses.

3. Please provide sequences or accession numbers for the SARS-CoV-2 antigens for S protein and RBD used in the study.

Vectors were received from Dr Florian Krammer. Added to Acknowledgement as resources that also gives accession number from those publications referenced:

We would like to thank Dr Florian Krammer at Mount Sinai Icahn School of Medicine for providing the mammalian expression vectors for the modified S protein and the RBD protein (9,10), which were produced under HHSN272201400008C and obtained through BEI Resources, NIAID, NIH: Spike

Glycoprotein Receptor Binding Domain (RBD) from SARS-Related Coronavirus 2, Wuhan-Hu-1 with C-Terminal Histidine Tag, Recombinant from HEK293F Cells, NR-52366 and Spike Glycoprotein (Stabilized) from SARS-Related Coronavirus 2, Wuhan-Hu-1 with C-Terminal Histidine Tag, Recombinant from Baculovirus, NR-52308.

4. For studies involving humans categorized by race/ethnicity, age, disease/disabilities, religion, sex/gender, sexual orientation, or other socially constructed groupings, authors should:

1) Explicitly describe their methods of categorizing human populations,

2) Define categories in as much detail as the study protocol allows,

3) Update outmoded terms and potentially stigmatizing labels to more current, acceptable terminology.

Examples: “Caucasian” should be changed to “white”

For this study, donors who presented to Hoxworth Blood Center were in 6 racial categories; Asian, Black or African American, Native Hawaiian or Other Pacific Islander, White, Other, or Non-specified as delineated per NIH Notice Number: NOT-OD-15-089

5. Thank you for stating the following in the Financial Disclosure section:

"This study was supported the Howard Hughes Medical Institute (to J.D.M. No grant number, Molkentin Investigator). J.D.M was also supported by an internal grant from Cincinnati Children Research Foundation to conduct these studies on COVID-19 (Molkentin grant 1). Contributions to this work by P.S. were financially assisted by The John Hauck Foundation, Fifth Third Bank.

https://www.cincinnatichildrens.org/

https://fconline.foundationcenter.org/fdo-grantmaker-profile?key=HAUC002

https://www.hhmi.org/

The funders had no role in study design, data collection and analysis, decision to publish, or preparation of the manuscript."

We note that you received funding from a commercial source: Fifth Third Bank.

Please provide an amended Competing Interests Statement that explicitly states this commercial funder, along with any other relevant declarations relating to employment, consultancy, patents, products in development, marketed products, etc.

Within this Competing Interests Statement, please confirm that this does not alter your adherence to all PLOS ONE policies on sharing data and materials by including the following statement: "This does not alter our adherence to PLOS ONE policies on sharing data and materials.” (as detailed online in our guide for authors http://journals.plos.org/plosone/s/competing-interests). If there are restrictions on sharing of data and/or materials, please state these. Please note that we cannot proceed with consideration of your article until this information has been declared.

Please include your amended Competing Interests Statement within your cover letter. We will change the online submission form on your behalf.

Please know it is PLOS ONE policy for corresponding authors to declare, on behalf of all authors, all potential competing interests for the purposes of transparency. PLOS defines a competing interest as anything that interferes with, or could reasonably be perceived as interfering with, the full and objective presentation, peer review, editorial decision-making, or publication of research or non-research articles submitted to one of the journals. Competing interests can be financial or non-financial, professional, or personal. Competing interests can arise in relationship to an organization or another person. Please follow this link to our website for more details on competing interests: http://journals.plos.org/plosone/s/competing-interests

The Fifth Third band reference for support was inaccurate and this has been removed in the revised manuscript. All other declarations are now made as requested in the sections "Competing interests" and "Data availability". We have also updated the "Sources of funding" section as requested with the website information, and proper declarations. The other requested information has been added to the cover letter to provide the requested assurances and to provide assurances as to the lack of "Competing interests". We have declared that no competing interests exist with this current work (all authors).

6. We noted in your submission details that a portion of your manuscript may have been presented or published elsewhere. [https://www.medrxiv.org/content/10.1101/2021.03.11.21253263v1.supplementary-material] Please clarify whether this publication was peer-reviewed and formally published. If this work was previously peer-reviewed and published, in the cover letter please provide the reason that this work does not constitute dual publication and should be included in the current manuscript.

As requested this is now mentioned in the cover letter and we state that this current work has not been peer-reviewed and formally published, although an earlier version of this work was uploaded to the medRxiv preprint server as a preliminary manuscript, as the editor indicates. This work does not constitute a dual publication because the preprint server version of the paper is not an official publication and it was not peer-reviewed. We make mention of this fact in the "Competing interest" section of the revised paper.

7. We note that you have indicated that data from this study are available upon request. PLOS only allows data to be available upon request if there are legal or ethical restrictions on sharing data publicly. For information on unacceptable data access restrictions, please see http://journals.plos.org/plosone/s/data-availability#loc-unacceptable-data-access-restrictions.

In your revised cover letter, please address the following prompts:

a) If there are ethical or legal restrictions on sharing a de-identified data set, please explain them in detail (e.g., data contain potentially identifying or sensitive patient information) and who has imposed

them (e.g., an ethics committee). Please also provide contact information for a data access committee, ethics committee, or other institutional body to which data requests may be sent.

We now include an Excel spreadsheet with as Supportive data that includes all the raw demographic data for the de-identified donor samples used in this study. This information is included in the cover letter as requested. Hence all raw and primary data are shared, and there is no full gel files of concern that is not already contained within the primary data (S1 Fig)

b) If there are no restrictions, please upload the minimal anonymized data set necessary to replicate your study findings as either Supporting Information files or to a stable, public repository and provide us with the relevant URLs, DOIs, or accession numbers. Please see http://www.bmj.com/content/340/bmj.c181.long for guidelines on how to de-identify and prepare clinical data for publication. For a list of acceptable repositories, please see http://journals.plos.org/plosone/s/data-availability#loc-recommended-repositories.

We will update your Data Availability statement on your behalf to reflect the information you provide.

Minimum anonymized data set is provided.

8. PLOS ONE now requires that authors provide the original uncropped and unadjusted images underlying all blot or gel results reported in a submission's figures or Supporting Information files. This policy and the journal's other requirements for blot/gel reporting and figure preparation are described in detail at https://journals.plos.org/plosone/s/figures#loc-blot-and-gel-reporting-requirements and https://journals.plos.org/plosone/s/figures#loc-preparing-figures-from-image-files. When you submit your revised manuscript, please ensure that your figures adhere fully to these guidelines and provide the original underlying images for all blot or gel data reported in your submission. See the following link for instructions on providing the original image data: https://journals.plos.org/plosone/s/figures#loc-original-images-for-blots-and-gels.

In your cover letter, please note whether your blot/gel image data are in Supporting Information or posted at a public data repository, provide the repository URL if relevant, and provide specific details as to which raw blot/gel images, if any, are not available. Email us at plosone@plos.org if you have any questions.

There is no full gel file of concern that is not already contained within the primary data (S1 Fig), hence Supporting Information is not needed. Indeed, the only gel image is from S1 Fig B and it is already the full raw data of the entire gel (not cropped), which is readily provided. All figures are in Tiff format as requested

9. We note that you have included the phrase "data not shown" in your manuscript. Unfortunately, this does not meet our data sharing requirements. PLOS does not permit references to inaccessible data. We require that authors provide all relevant data within the paper, Supporting Information files, or in an acceptable, public repository. Please add a citation to support this phrase or upload the data that

corresponds with these findings to a stable repository (such as Figshare or Dryad) and provide and URLs, DOIs, or accession numbers that may be used to access these data. Or, if the data are not a core part of the research being presented in your study, we ask that you remove the phrase that refers to these data.

The reference to data not shown has been removed and we have modified this sentence and provided the necessary reference in the revised manuscript. Line 211-213 ELISA comparison between our ELISA and Krammer (9,10).

10. Your ethics statement should only appear in the Methods section of your manuscript. If your ethics statement is written in any section besides the Methods, please move it to the Methods section and delete it from any other section. Please ensure that your ethics statement is included in your manuscript, as the ethics statement entered into the online submission form will not be published alongside your manuscript.

Ethics statement has been moved to MM.

11. We note that Figure 2 in your submission contain map images which may be copyrighted. All PLOS content is published under the Creative Commons Attribution License (CC BY 4.0), which means that the manuscript, images, and Supporting Information files will be freely available online, and any third party is permitted to access, download, copy, distribute, and use these materials in any way, even commercially, with proper attribution. For these reasons, we cannot publish previously copyrighted maps or satellite images created using proprietary data, such as Google software (Google Maps, Street View, and Earth). For more information, see our copyright guidelines: http://journals.plos.org/plosone/s/licenses-and-copyright.

We require you to either (1) present written permission from the copyright holder to publish these figures specifically under the CC BY 4.0 license, or (2) remove the figures from your submission:

The original figure has been removed from the paper and an open source similar to the original has been added.

11.1. You may seek permission from the original copyright holder of Figure 2 to publish the content specifically under the CC BY 4.0 license.

We recommend that you contact the original copyright holder with the Content Permission Form (http://journals.plos.org/plosone/s/file?id=7c09/content-permission-form.pdf) and the following text:

“I request permission for the open-access journal PLOS ONE to publish XXX under the Creative Commons Attribution License (CCAL) CC BY 4.0 (http://creativecommons.org/licenses/by/4.0/). Please be aware that this license allows unrestricted use and distribution, even commercially, by third parties. Please reply and provide explicit written permission to publish XXX under a CC BY license and complete the attached form.”

Please upload the completed Content Permission Form or other proof of granted permissions as an "Other" file with your submission.

In the figure caption of the copyrighted figure, please include the following text: “Reprinted from [ref] under a CC BY license, with permission from [name of publisher], original copyright [original copyright year].”

11.2. If you are unable to obtain permission from the original copyright holder to publish these figures under the CC BY 4.0 license or if the copyright holder’s requirements are incompatible with the CC BY 4.0 license, please either i) remove the figure or ii) supply a replacement figure that complies with the CC BY 4.0 license. Please check copyright information on all replacement figures and update the figure caption with source information. If applicable, please specify in the figure caption text when a figure is similar but not identical to the original image and is therefore for illustrative purposes only.

The following resources for replacing copyrighted map figures may be helpful:

USGS National Map Viewer (public domain): http://viewer.nationalmap.gov/viewer/

The Gateway to Astronaut Photography of Earth (public domain): http://eol.jsc.nasa.gov/sseop/clickmap/

Maps at the CIA (public domain): https://www.cia.gov/library/publications/the-world-factbook/index.html and https://www.cia.gov/library/publications/cia-maps-publications/index.html

NASA Earth Observatory (public domain): http://earthobservatory.nasa.gov/

Landsat: http://landsat.visibleearth.nasa.gov/

USGS EROS (Earth Resources Observatory and Science (EROS) Center) (public domain): http://eros.usgs.gov/#

Natural Earth (public domain): http://www.naturalearthdata.com/

The Map that is now provided was adapted from http://viewer.nationalmap.gov/viewer/ and is public domain.

Citation:

USGS the national Map: National Boundaries Dataset, 2DEP Elevation Program, Geographical Names Information System, National Hydrography Dataset, National land Cover Database, national Structures, dataset, and National Transportation Dataset; USGS Global Ecosystem; U.S. Census Bureau TIGER/Line data; USFS Road Data; Natural Earth data; U.S. Department of State Humanitarian Information Unit; and NOAA National Centers for Environmental Information, US Coastal Relief Model. Data refreshed May, 2020.

12. Please include captions for your Supporting Information files at the end of your manuscript, and update any in-text citations to match accordingly. Please see our Supporting Information guidelines for more information: http://journals.plos.org/plosone/s/supporting-information.

This has been added.
